# Isolation and characterization of three new anti-proliferative Sesquiterpenes from *Polygonum barbatum* and their mechanism via apoptotic pathway

**DOI:** 10.1186/s12885-017-3667-9

**Published:** 2017-10-23

**Authors:** Umar Farooq, Sadia Naz, Binte Zehra, Ajmal Khan, Syed Abid Ali, Ayaz Ahmed, Rizwana Sarwar, Syed Majid Bukhari, Abdur Rauf, Izhar Ahmad, Yahia Nasser Mabkhot

**Affiliations:** 1 Department of Chemistry, COMSATS Institute of Information Technology, Abbottabad, KPK 22060 Pakistan; 2 Dr. Panjwani Center for Molecular Medicine and Drug Research, International Center for Chemical and Biological Sciences, University of Karachi, Karachi, 75270 Pakistan; 3 H.E.J. Research Institute of Chemistry, International Center for Chemical and Biological Sciences, University of Karachi, Karachi, 75270 Pakistan; 4 Department of Chemistry, University of Swabi, Anbar, Khyber Pakhtunkhwa 23561 Pakistan; 5 Department of Botany, Islamia College Peshawar, Peshawar, Pakistan; 6 Department of Chemistry, College of Science, King Saud University, P. O. Box 2455, Riyadh, 11451 Saudi Arabia

**Keywords:** *Polygonum Barbatum*, Sesquiterpenes, Non-small cell lung carcinoma, Angiogenesis, VEGF, Cox-2, Apoptosis

## Abstract

**Background:**

The emergence of chemoresistant cancers and toxicity related to existing chemotherapeutic agents, demand the search for new pharmacophore with enhanced anti-cancer activity and least toxicity. For this purpose, three new sesquiterpenes were isolated from ethyl acetate fraction of the aerial parts of the plant *Polygonum barbatum* and evaluated for their anti-cancer potential.

**Methods:**

The structural elucidation and characterization of the isolated compounds 1–3 were performed using various spectroscopic techniques such as mass, UV, IR, and extensive 1D/2D–NMR spectroscopy. Furthermore, the compounds 1–3 were subjected to screening of anti-cancer activity against different cell lines followed by brief analysis of apoptotic and anti-angiogenic potentials of the potent hit against non-small cell lung carcinoma cell line.

**Results:**

All the compounds 1–3 were subjected to anti-proliferative potential against non-small cell lung carcinoma (NCI-H460), breast cancer (MCF-7), cervical cancer (HeLa) and normal mouse fibroblast (NIH-3 T3) cell lines. Among these, compound 3 was found to be more cytotoxic against NCI-H460 and MCF-7 cells (IC_50_ = 17.86 ± 0.72 and 11.86 ± 0.46 μM respectively). When compared with the standard drug cisplatin compound 3 was found to have more potent activity against NCI-H460 (IC_50 =_ 19 ± 1.24 μM) as compared to MCF-7 cell lines (IC_50 =_ 9.62 ± 0.5 μM). Compound 3 induced apoptosis in NCI-H460 cells in a dose dependent manner. It significantly downregulated, the expression of anti-apoptotic (BCL-2 L1 and p53) and increased the expression of pro-apoptotic (BAK and BAX) genes. Besides apoptosis, it also significantly reduced the cell migration and downregulated the angiogenic genes (i.e. VEGF and COX-2), thereby, inhibiting angiogenesis in NCI-H460 cells.

**Conclusion:**

Compound 3 possesses potent anti-proliferative potential as well as induced apoptosis and inhibited the cell migration of the cancerous cells by altering the gene expression, responsible for it.

## Background

*Polygonum barbatum* is a herbaceous perennial weed commonly known as “Knot weed” belonging to Polygonaceae, which grows mostly in shady and moist areas, along the sides of rivers and ponds [1]. The family Polygonaceae comprises of 50 genera and ~1200 species widely distributed in Asia and America, and is represented in Pakistan by 19 genera and 103 species [2]. *Polygonum barbatum* is used as traditional medicine; the leaf extract of *Polygonum barbatum* has been used for the treatment of ulcers, while its roots are used as an astringent traditionally by local practitioners [3]. According to literature survey, *Polygonum barbatum* possesses cholinergic, antinociceptive, anti-tumor, anti-inflammatory, antivenom and diuretic activities [4]. In addition, brine shrimp toxicity and spasmolypic activity of its dichloromethane extract has also been reported previously [5].

Various secondary metabolites, such as flavonoids, anthraquinones, phenylpropanoids and proanthocyanidins have been reported from various species of the genus *Polygonum* [6–8]. The bioactive constituents of *Polygonum barbatum*, including sitosterone, viscozulenic acid and acetophenone, have also been reported previously [1]. Natural products, because of their excellent bioavailability and abundance are getting much attention in cancer therapy for the last few decades as sources to find new and novel drug alternates.

Globally, cancers are the leading cause of death after cardiovascular diseases. Among various cancers, non-small cell lung carcinoma (NSCLC) is the most common type of lung cancer occurring worldwide with high rates of morbidity and mortality. Lung cancer alone accounts for one-third of the cancer related mortality across the world. Excessive tobacco use makes the lung cancer as the third most prevalent disease in Pakistan. The advancement of therapeutic regimen increased the survival rate to about 65%, however, cancer progression, metastatic potential and chemoresistance still are the major concerns associated with high mortality rates [9]. Modern lifestyle, smoking habits and occupational exposures to the chemicals such as asbestos, uranium and coke are the contributing factors responsible for the initiation and progression of cancer.

Tumor formation is a complex process, which involves sustained growth signals, inhibition of growth suppressors, preventing cellular death, angiogenesis, invasion and spread to the secondary sites [10]. Tumor mass is known to emerge from normal cells after mutation, which then deviates from their normal cycle of homeostasis, survival and death [11]. It starts as a single tumor cell, which after interaction with nearby vascular components, develops and results in neoplasm with altered gene expression [12]. Lack of apoptosis, migration and invasion are the hallmarks of cancer. Apoptosis is an orderly cell death in response to any damage caused to cell’s basic functionality. It begins as a cascade of molecular events, eventually resulting in the cellular death and phagocytosis to prevent inflammation [13]. A number of apoptotic markers including tumor suppressor proteins, caspases, BCL-2 proteins and several others are known to be crucial for the process [14]. Studies have shown that apoptosis could occur through any of the two pathways (intrinsic or extrinsic) depending upon the initiating signals. Both pathways converge to the activation of effector caspases, which leads to apoptosis [15].

Angiogenesis plays an important role in the advancement of cancer. Many pro-angiogenic factors work in association with each other to provide vasculature to the newly formed mass [16]. Vascular epithelial growth factor (VEGF) is the major angiogenic protein that stimulates the growth of vascular endothelial cells. Many cancers including breast, prostate and lung cancers secrete high levels of this mitogen [17, 18]. While cyclooxygenase-2 (COX-2) is an enzyme, which converts arachidonic acid to prostaglandin H2 (PGH2). The PGH2 are acted upon by prostaglandin synthase E to produce prostaglandin E, which results in tumor invasion [19, 20].

The emergence of chemoresistant cancer and toxic effects of existing pharmacophore, demand the search for the alternates with potent anti-cancer activity and less toxicity. In the present study, we are reporting the isolation, structure elucidation and the anticancer activities of three new sesquiterpenes 1–3 from aerial parts of the *Polygonum barbatum* against non-small cell lung carcinoma (NCI-H640), breast cancer (MCF-7), cervical cancer (HeLa) and normal mouse fibroblast (NIH-3 T3) cell lines. These cell lines were selected on the basis of its availability as well as its prevalence in Pakistani population. Moreover, in depth analysis of the active compounds were further evaluated against the respective cell lines.

## Results

The ethyl acetate fraction of *Polygonum barbatum* was subjected to column chromatography on silica gel packed columns. The repeated column chromatography resulted in the isolation of three new sesquiterpenes derivatives (1–3); their characterization was done by using various spectroscopic techniques as well as through literature comparison.

### Compound 1

Compound 1 was isolated as a brownish gum having molecular formula C_16_H_18_O_4_ based on molecular ion peak at m/z 274.1211 in HR-EI-MS, while the fragmentations were found at m/z (%)256 (50), 242 (75), 238 (65), 227 (60), 192 (70), 188 (30), 151 (100), 126 (80) and 93 (40). The UV spectrum showed absorption bands at λ_max_ 220 (2.8), 272 (3.6), 314 (4.3) and 336 cm^−1^ (2.9), while the IR spectrum showed absorptions at 3398, 3075, 2982, 1718, 1630 indicating the presence of hydroxyl group, ketone and aromatic moiety, respectively.

The ^1^H NMR spectrum showed the presence of two aromatic protons at δ_H_ 7.22 (1H, d, *J* = 9.0 Hz) and δ_H_ 7.46 (1H, d, *J* = 9.0 Hz) *ortho* coupled to each other along with one methylene singlet at δ_H_ 4.99 (2H, s), a multiplet for one methine proton at δ_H_ 3.20 (1H, m) and a singlet for three methoxy protons at δ_H_ 3.90 (3H, s). Similarly, two olefinic protons at chemical shift values of δ_H_ 6.80 (1H, s) and δ_H_ 6.51 (1H, m) and a methyl doublet integrating for six protons, appeared at δ_H_ 1.26 (6H, d, *J* = 7.4 Hz) in ^1^H NMR spectrum (Table 1).

**Table 1:** ^1^H NMR (500 MHz, CDCl_3_), δ_H_ in ppm

	Compound 1	Compound 2	Compound 3
Carbon No	^1^H–NMR (δ_H_ ppm)	^1^H–NMR (δ_H_ ppm)	^1^H–NMR (δ_H_ ppm)
1	–	–	–
2	–	–	–
3	6.80, s	6.83, s	6.75, s
4	–	–	–
5	–	–	–
6	7.22 (d, *J* = 9.0 Hz)	7.20 (d, *J* = 8.3 Hz)	7.22 (d, *J* = 9.0 Hz)
7	7.46 (d, *J* = 9.0 Hz)	7.28 (d, *J* = 8.3 Hz)	7.48 (d, *J* = 9.0 Hz)
8	–	–	–
9	–	–	–
10	6.51, (d, *J* = 11.1 Hz)	6.48, (d, *J* = 11.0 Hz)	6.50, (d, *J* = 10.8 Hz) m
11	3.20, m	3.20, m	3.18, m
12	1.26, (d, *J* = 7.4 Hz)	1.28, (d, *J* = 7.1 Hz)	1.29, (d, *J* = 7.8 Hz)
13	1.26, (d, *J* = 7.4 Hz)	1.28, (d, *J* = 7.1 Hz)	1.29, (d, *J* = 7.8 Hz)
14	–	–	–
15	4.99, s	–	–
16	–	2.40, s	2.43, s
9-OCH_3_	3.90, s	3.88, s	3.80, s
14-OCH_3_	–	–	3.87, s

**Fig. 1:**
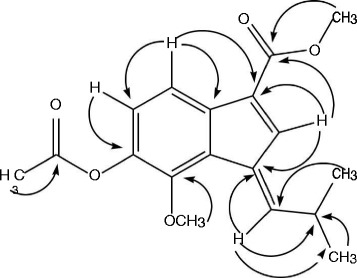
Important HMBC (→) correlations of compound 1–3

The ^13^C NMR and DEPT spectra revealed the presence of 16 carbon atoms including three methyls, five methines, one methylene, and seven quaternary carbons. Two methyl carbons being identical resonated at δ_C_ 27.6, while the methoxy group reappeared at δ_C_ 58.7 and methylene carbon having hydroxyl group as substituent centered at δ_C_ 62.4 (Table 2). The ^13^C NMR spectrum showed presence of five methine carbons at δ_C_ 32.9, 143.2, 132.3, 119.7, 133.4 ppm. Similarly, one carbonyl carbon at δ_C_ 179.8 along with other quaternary carbons resonating at δ_C_ 138.1, 139.6, 123.9, 158.5, 129.7, and 140.1 were also observed in ^13^C NMR spectrum. The HMBC spectrum showed correlation of H-3 with C-1, C-2, C-4, C-5, while proton present at position H-6 showed strong HMBC correlation with C-1, C-5, C-7, C-8 and C-9 indicating the presence of indene ring in compound 1 [21]. The placement of substituents on this indene ring were also established from HMBC spectrum like methylpropylidine substituent was placed at position 2 of indene ring on the basis of strong HMBC correlation of olefinic proton at position H-10 with C-2, C-1 and C-3 along with HMBC correlation of other olefinic proton at position H-3 with C-2, C-4 and C-10. The position of other substituents like carboxylic group, methoxy group and methyl alcohol moiety were also confirmed on the basis of HMBC spectrum as depicted in Fig. 1. The structure of compound 1 was suggested to be (*E*)-6-(hydroxymethyl)-7-methoxy-1-(2-methylpropylidene)-1H–indene-3-carboxylic acid on the basis of above mentioned spectral data as well as comparison with literature.

**Table 2:** ^13^C NMR (125 MHz, CDCl_3_), δ_C_ in ppm

	Compound **1**	Compound **2**	Compound **3**
Carbon No	^13^C–NMR (δ_C_ ppm)	^13^C–NMR (δ_C_ ppm)	^13^C–NMR (δ_C_ ppm)
1	123.9	123.2	125.3
2	138.1	140.4	140.7
3	132.3	130.7	122.6
4	140.1	138.8	134.7
5	139.6	137.7	138.4
6	119.7	120.1	120.1
7	133.4	125.4	128.1
8	129.7	144.7	146.7
9	158.5	160.1	159.6
10	143.2	141.4	142.1
11	32.9	32.4	32.1
12	27.6	28.3	25.4
13	27.6	28.3	25.4
14	179.8	180.7	175.1
15	62.4	174.1	172.6
16	–	23.4	22.7
9-OCH_3_	58.7	60.4	59.6
14-OCH_3_	–	–	57.6

### Compound 2

Compound 2 was isolated as brownish gummy solid from ethyl acetate fraction of *Polygonum barbatum*. The HR-EI-MS showed molecular ion peak at m/z 302.1160 suggesting a molecular formula C_17_H_18_O_5_ for Compound 2 and m/z (%) other fragments ion peaks were observed at 284 (70), 252 (65), 238 (40), 194 (57), 166 (100), 151 (90), 124 (75) and 93 (20). The UV spectrum revealed absorptions bands at λ_max_ 218 (3.6), 274 (4.2), 316 (3.7) and 328 (2.9), while the IR spectrum showed peaks at 3460, 3009, 1752, 1705, 1640 cm^−1^ were quite similar to that of compound 1 suggesting the presence of hydroxyl moiety, alkene, ketone and aromatic groups in compound 2.

All the spectroscopic data like; ^1^H NMR and ^13^C NMR of compound 2 is quite identical to compound 1 having two aromatic proton along their ^13^C NMR appeared at δ_H_ 7.20 (1H, d, *J* = 8.3 Hz, δ_C_ 120.1) and δ_H_ 7.28 (1H, d, *J* = 8.3 Hz, δ_H_ 125.4) showed *ortho* coupling with each other, while the two olefinic protons at δ_H_ 6.82 (1H, s, δ_C_ 141.4) and δ_H_ 6.48 (1H, m, δ_C_ 130.7) presenting two pair of double bonds (C-2―C-4, C-10). Two identical methyl group appeared at δ_H_ 1.28 (6H, d, *J* = 7.1, δ_C_ 28.3) and protons of methoxy group gave singlet at δ_H_ 3.88 (3H, s, δ_C_ 60.4) suggesting presence of indene ring similar to compound 1 [21]. The only difference observed was the presence of ester group at position 8 of indene ring protons of compound 2, where methyl of ester moiety appeared as singlet at δ_H_ 2.40 (3H, s, δ_C_ 23.4).

The position of substituents at indene ring of compound 2 was established through HMBC correlations almost similar to compound 1, the only difference observed was presence of ester group at position 8. Finally, the structure of compound 2 on the basis of all the above spectroscopic data and comparison with literature was as (*E*)-6-acetoxy-7-methoxy-1-(2-methylpropylidene)-1H–indene-3-carboxylic acid.

### Compound 3

Compound 3 was isolated as light brown gummy solid with molecular formula C_18_H_20_O_5_ as suggested by molecular ion peak at m/z 316.1320 in HR-EI-MS (calcd. 316.1311). The other fragmentation peaks were observed at m/z (%) 285 (40), 256 (55), 225 (63), 196 (75), 168 (70), 154 (100), 128 (80) and 94 (35). The UV spectrum showed absorption bands at λ_max_ 222 (4.3), 270 (3.8), 318 (3.1) and 332 (3.6) while IR spectrum showed absorption bands at 3390, 2960, 1746, 1676 br and 1560 cm^−1^ similar to compound 1 and 2.

The spectroscopic data obtained from both ^1^H NMR and ^13^C NMR were similar to compound 1 and 2 with two olefinic protons resonated at δ_H_ 6.75 (1H, s, δ_C_ 122.6) and δ_H_ 6.50 (1H, m, δ_C_ 142.1), while two aromatic protons were also observed at δ_H_ 7.22 (1H, d, *J* = 9.0 Hz, δ_C_ 120.1) and δ_H_ 7.48 (1H, d, *J* = 9.0 Hz, δ_C_ 128.1). Protons of two methoxy groups appeared as singlet at δ_H_ 3.80 (3H, s, δ_C_ 59.6) and 3.87 (3H, s, δ_C_ 57.6), while two methyl group resonated at δ_H_ 1.29 (6H, d, *J* = 7.8 Hz, δ_C_ 25.4) and another methyl group directly connected to carbonyl carbon appeared at δ_H_ 2.43 (3H, s, δ_C_ 22.7). The ^13^C NMR spectra revealed the presence of 18 carbon atoms, while HMBC correlations were quite helpful for placement of substituents on basic skeleton (indene ring). The appearance of one methoxy group 3.87 (3H, s, δ_C_ 57.6) signals and the disappearance of one hydroxyl group were observed in compound 3. Further analysis of its HMBC correlations suggested the methoxy group located at C-14. All spectral data of compound 3 was found similar to compound 2 the only difference observed was extra methoxy group attached to carbonyl carbon at position 14, further supported by mass fragmentation pattern and structure assigned to compound 3 was (*E*)-methyl 6-acetoxy-7-methoxy-1-(2-methylpropylidene)-1H–indene-3-carboxylate.

### Characterization of compound 1

A brownish gum; UV (MeOH) λ_max_ 220 (2.8), 272 (3.6), 314 (4.3), 336 (2.9) nm; IR (KBr) ʋ_max_ 3398, 3075, 2982, 1718, 1630, 1540, 1160, 880, 793 cm^−1^; EI-MS m/z (%): 256 (50), 242 (75), 238 (65), 227 (60), 192 (70), 188 (30), 151 (100), 126 (80), 93 (40); HR-EI-MS: m/z [M]^+^ Calcd. 274.1205 for C_16_H_18_O_4_; found 274.1211, ^1^H NMR (500 MHz, CDCl_3_) δ (ppm): 6.80 (H-3, s), 7.22 (H-6, d, *J* = 9.0 Hz), 7.46 (H-7, d, *J* = 9.0 Hz), 6.51 (H-10, (d, *J* = 11.1 Hz), 3.20 (H-11, m), 1.26 (H-12/13, d, *J* = 7.4 Hz), 4.99 (H-15, s), 3.90 (9-OMe, s); ^13^C NMR (125 MHz, CDCl_3_) δ (ppm): 123.9 (C-1), 138.1 (C-2), 132.3 (C-3), 140.1 (C-4), 139.6 (C-5), 119.7 (C-6), 133.4 (C-7), 129.7 (C-8), 158.5 (C-9), 143.2 (C-10), 32.9 (C-11), 27.6 (C-12/13), 179.8 (C-14), 62.4 (C-15), 58.7 (9-OMe).

### Characterization of compound 2

A brownish gum; UV (MeOH) λ_max_ 218 (3.6), 274 (4.2), 316 (3.7), 328 (2.9) nm; IR (KBr) ʋ_max_ 3460, 3009, 2982, 1752, 1705, 1640, 1570, 1480, 1176, 910 cm^−1^; EI-MS m/z (%): 284 (70), 252 (65), 238 (40), 194 (57), 166 (100), 151 (90), 124 (75), 93 (20); HR-EI-MS: m/z [M]^+^ Calcd. 302.1154 for C_17_H_18_O_5_; found 302.1160, ^1^H NMR (500 MHz, CDCl_3_) δ_H_ (ppm): 6.82 (H-3, s), 7.20 (H-6, d, *J* = 8.3 Hz), 7.28 (H-7, d, *J* = 8.3 Hz), 6.48 (H-10, (d, *J* = 11.0 Hz), 3.20 (H-11, m), 1.28 (H-12/13, d, *J* = 7.1 Hz), 2.40 (H-16, s), 3.88 (9-OMe, s); ^13^C NMR (125 MHz, CDCl_3_) δ_C_ (ppm): 123.2 (C-1), 140.4 (C-2), 130.7 (C-3), 138.8 (C-4), 137.7 (C-5), 120.1 (C-6), 125.4 (C-7), 144.7 (C-8), 160.1 (C-9), 141.1 (C-10), 32.4 (C-11), 28.3 (C-12/13), 180.7 (C-14), 174.1 (C-15), 23.4 (C-16), 60.4 (9-OMe).

### Characterization of compound 3

A light brown gummy solid; UV (MeOH) λ_max_ 222 (4.3), 270 (3.8), 318 (3.1), 332 (3.6) nm; IR (KBr) ʋ_max_ 3390, 2960, 1746, 1676 br, 1560, 1330 cm^−1^; EI-MS m/z (%): 285 (40), 256 (55), 225 (63), 196 (75), 168 (70), 154 (100), 128 (80), 94 (35); HR-EI-MS: m/z [M]^+^ Calcd. 316.1311 for C_18_H_20_O_5_; found 316.1320, ^1^H NMR (500 MHz, CDCl_3_) δ_H_ (ppm): 6.75 (H-3, s), 7.22 (H-6, d, *J* = 9.0 Hz), 7.48 (H-7, d, *J* = 9.0 Hz), 6.50 (H-10, (d, *J* = 10.8 Hz), 3.18 (H-11, m), 1.29 (H-12/13, d, *J* = 7.8 Hz), 2.43 (H-16, s), 3.80 (9-OMe, s), 57.6 (14-OMe); ^13^C NMR (125 MHz, CDCl_3_) δ_C_ (ppm): 125.3 (C-1), 140.7 (C-2), 122.6 (C-3), 134.7 (C-4), 138.4 (C-5), 120.1 (C-6), 128.1 (C-7), 146.7 (C-8), 159.6 (C-9), 142.1 (C-10), 32.1 (C-11), 25.4 (C-12/13), 175.1 (C-14), 57.6 (14-OMe), 172.6 (C-15), 22.7 (C-16), 59.6 (9-OMe).

### Effects of compound 1–3 on proliferation and survival of cancer cell lines

Reduction of viable cells after the treatment with compounds 1–3 was determined by MTT assay. Cytotoxicity of these compounds was evaluated against human non-small cell lung carcinoma (NCI-H460), MCF-7 (breast cancer), HeLa (cervical cancer) and normal mouse fibroblast cells (NIH-3 T3). Among all cell lines, the three sesquiterpenes showed anti-cancer activity against cancer cells and was less active against normal 3 T3 cells. However, compound 3 was found to be potentially anti-proliferative in a dose dependent manner against NSCLC cells when compared to compounds 1–2 and the standard drug cisplatin (Table 3). Compound 3 was also found to be less cytotoxic against normal cells as compared to lung cancer cells. By considering its higher anti-cancer and selective toxicity towards cancer cells at a lower concentration as compared to normal cells, it was selected for further detailed studies against NCI-H460 cells.

**Table 3:** 50% inhibitory concentration of compound 1–3 against various cell lines. The compound 3 showed potent anti-cancer activity against NSCLC cells as compared to cisplatin (standard drug)

	NCI-H460	MCF-7	HeLa	NIH-3 T3
Compound 1	0.23 mM ± 0.85	Inactive	Inactive	0.42 mM ± 0.13
Compound 2	0.13 mM ± 0.38	Inactive	Inactive	0.28 mM ± 0.5
Compound 3	17.86 μM ± 0.72	11.86 μM ± 0.46	32.13 μM ± 0.6	30.32 μM ± 0.93
Cisplatin	19 μM ± 1.24	09.62 μM ± 0.5	16.19 μM ± 0.7	–

**Fig. 2:**
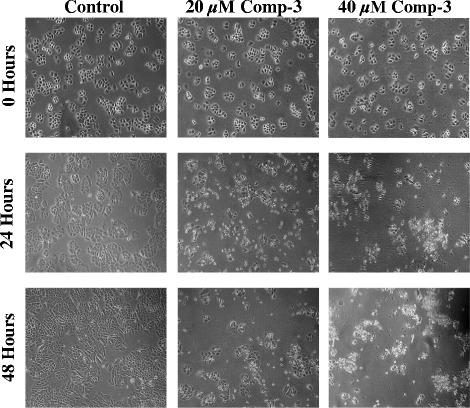
Phase contrast microscopic images of NCI-H460 cells after treatment with compound 3 at 20 and 40 μM concentrations. In control well, the cells were in their normal morphology while treated cells died and escaped the monolayer after 24 h of the treatment. The images were taken at 10X magnification

**Fig. 3:**
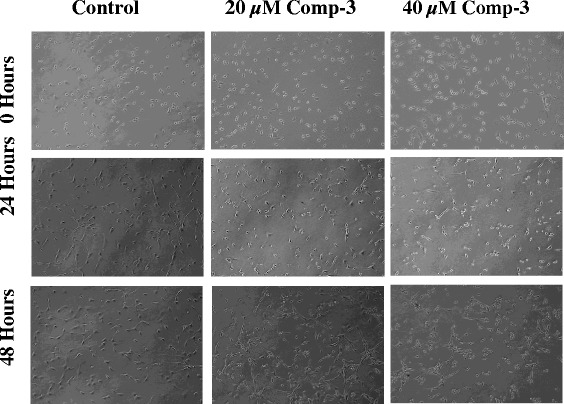
Phase contrast microscopic images of normal NIH-3 T3 cells after treatment with compound 3 at 20 and 40 μM concentrations at different time intervals. Control well showed cells in their normal morphology while both live and dead populations were found in treated wells after 48 h. The images were taken at 10X magnification

The anti-proliferative potential of the compound 3 on H460 and 3 T3 cells was further confirmed by phase contrast microscopy. Images of cells were captured after treatment with compound 3 at 20 and 40 μM concentrations at 0, 24 and 48 h. After 24 h, H460 cells started to change their normal morphology and got detached from the monolayer, while after 48 h, majority of the cells died, formed clumps with other dead cells in the media as compared to vehicle treated cells (Fig. 2). Whereas, the same doses were applied to 3 T3 cells; inhibition was observed at both doses after 24 h of treatment but growing cells with normal morphology appeared after 48 h of the treatment: depicting compound’s selective toxicity against cancer cells (Fig. 3).

### Compound 3 induces apoptosis in NCI-H460 cells

**Fig. 4:**
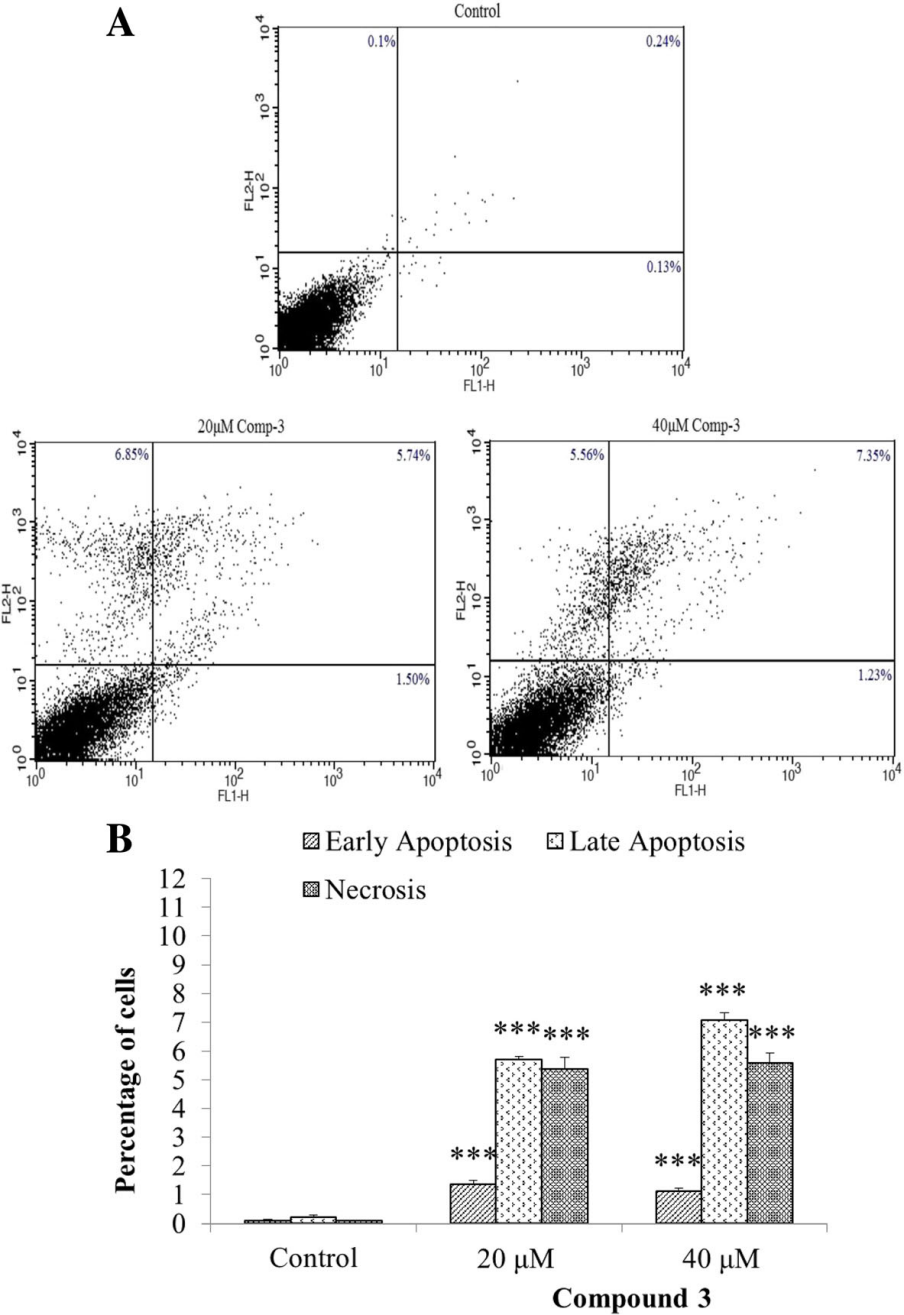
**a** The apoptotic potential of compound 3 in lung cancer NCI-H460 cells. FACS images showed percent apoptotic cells after treatment with compound 3. Cells were significantly undergoing apoptosis as compared to the vehicle control. **b** Graphical representation of the cell population in each phase of apoptosis and expressed as the mean of three independent experiments. *** *p* < 0.001 as compared to control

Phase contrast microscopy results showed the presence of necrotic or dead cells, after treating with compound 3. The extent of apoptosis and percent population of the cells in early, late apoptotic phases and necrosis were also analyzed. The population of non-treated cells was concentrated in lower left quadrant indicating the normal cells. On the other hand, treated cells showed significant apoptotic change with major cell density undergoing late phases of apoptosis (upper right quadrant) and necrosis (upper left quadrant) at 20 μM concentration, while at 40 μM, more cells were found to be in late apoptotic phase (Fig. 4). The overall percentage of apoptotic cells after treating with compound 3 at 20 and 40 μM was found to be around 7% and 8.5% respectively.

**Fig. 5:**
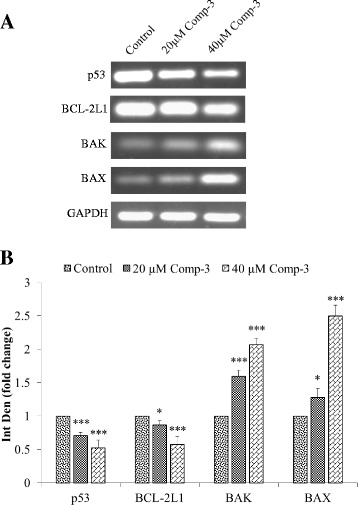
**a** Gene expression analysis of pro-apoptotic (BAK and BAX) and anti-apoptotic genes (BCL-2 L1 and p53) after 48 h treatment of NCI-H460 cells with compound 3. GAPDH was used as a control housekeeping gene. **b** Quantitative analysis of the expression by calculating fold change in integrated density treated versus control genes. *** *p* < 0.001 and ** p < 0.01 when compared to the control

The apoptotic ability of compound 3 was further confirmed by gene expression analysis of anti-apoptotic genes (BCL-2 L1 and p53), pro-apoptotic genes (BAK and BAX). As observed, the expression of anti-apoptotic genes BCL-2 L1 and p53 was significantly down regulated and expression of pro-apoptotic genes BAK and BAX was upregulated as compared to vehicle control after 48 h treatment (Fig. 5). GAPDH was used as constitutive gene and its expression remained constant in the treated cells, when compared to control.

### Anti-angiogenic potential of compound 3 on NCI-H460 cells

**Fig. 6:**
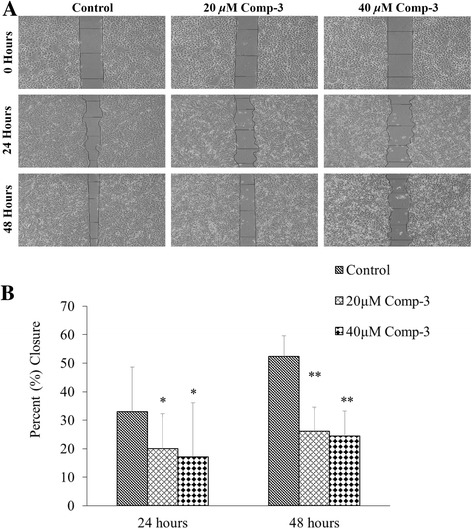
**a** Anti-migration potential of compound 3 at 20 and 40 μM concentrations against lung cancer NCI-H460 cells. The control cells healed 33% and 52% scratch after 24 h and 48 h whereas 17% and 24% healing was observed at 40 μM of compound 3. **b** Graphical representation of the rates of migration in control and treated wells. ** *p* < 0.01 and * p < 0.05 control vs treated

**Fig. 7:**
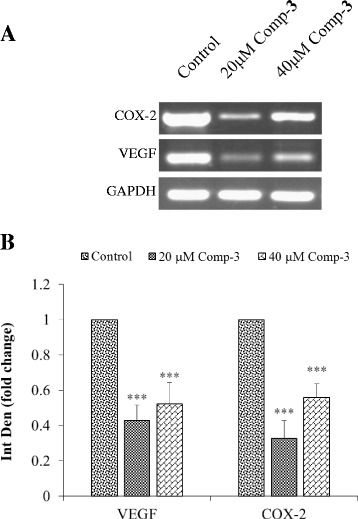
**a** Gene expression of angiogenic VEGF and COX-2 genes after the treatment of NCI-H460 cells. **b** Graphical representation of quantitative analysis of the gene expression. *** p < 0.001 as compared to the control

Angiogenesis is the most important processes during wound healing or cancer cell migration [22, 23]. Therefore, the compound 3 was also evaluated for its anti-migratory potential. Results showed that it significantly delayed the rate of wound healing at 20 and 40 μM concentrations as compared to untreated cells (Fig. 6). After 48 h, only 17% and 24% scratch were closed at 20 and 40 μM concentrations, respectively, as compared to vehicle treated control (52%). Gene expression of two important angiogenic markers i.e., VEGF and COX-2, responsible for potentiating the migration and invasion were also established (Fig. 7). Significant downregulation of both genes further links with the anti-angiogenic potential of compound 3. These compounds also alter the expression of matrix metalloproteinases in a dose dependent manner (data not shown). Interestingly, at 40 μM concentrations, there was a slight increase in the expression of genes as compared to 20 μM clearly suggesting a dose dependent phenomenon.

## Discussion

Cancer, being one of the emerging concerns of mortality worldwide, demands the alternative approaches to deal with it. Due to the toxicity of available drugs and emerging chemo-resistance, the medicinal plants can provide better alternates that could be developed into new pharmacophores with enhanced anticancer activity and less toxicity. In fact, 60% of the anti-cancer drugs (e.g. vinblastine, paclitaxel, etoposide, doxorubicin etc.) are derived from the natural origin [24, 25]. Thus, this study was designed to screen the anti-cancer potential of three novel sesquiterpenes isolated from the aerial parts of *Polygonum barbatum* on different cancer cell lines (Table 3). Compound 1 and 2 were found to be inactive against the tested cell lines. Whereas, compound 3 was found to be anti-proliferative against all tested cancer cell lines. In comparison to its activity against normal cells (3 T3), higher anti-cancer activity was observed in NCI-H460 and MCF-7 cells than HeLa cells. A possible explanation to this observation could be interference of tumor suppressor protein p53. All cell lines are positive for TP53 but it gets inactivated in HeLa cells due to human papilloma virus; HeLa cells contain HPV-18 sequences. While when compared to IC_50_ values of compound 3 and the standard drug, it was most potent against NCI-H460 cells. Hence, in-depth study of mechanism was evaluated against non-small cell lung carcinoma (NSCLC) cells.

Interestingly, compound 3 showed prominent anti-proliferative effects after 24 h of treatment as observed by phase contrast microscopy in H460 cells (Fig. 2). However, slight inhibition was also observed in treated wells of 3 T3 cells but the effect reverted after 48 h resulting in re-emergence of actively growing cells (Fig. 3). Compound 3 also induced apoptosis and inhibit cell migration in NCI-H460 cells after 24 and 48 h of treatment, and altered the genes responsible for apoptosis and migration of cancerous cells.

Apoptosis is a very vital maintenance system, which keeps a check on the unhealthy, malignant, dead or infected cells of the body. It functions in a regulatory manner and can be triggered by a variety of responses, including physiological or pathological or both [11]. The early phases of apoptosis correspond to the early events of activation of intrinsic or extrinsic pathways whereas events downstream the cleavage of caspases-3 encompasses the late phase of apoptosis [26]. Cancer cells take control of apoptotic machinery and promote tumour progression. Literature revealed that most of the existing drugs target apoptosis, which is crucial in cancer advancement [14, 27]. Our present results demonstrate that compound 3 also induced apoptosis in the treated cells. The FACS analysis by using dual staining with YO-PRO-1 and PI dyes revealed that after 48 h treatment, cells were found to be in late phases of apoptosis and underwent necrosis (Fig. 4).

Usually, a cell undergoes either intrinsic mitochondrial pathway or extrinsic receptor pathways to initiate the process of apoptosis. Absence of growth factors or cytokines, radiation, hypoxia, ROS and infections, results in initiation of mitochondrial pathway*.* Stimuli lead to a change in mitochondrial membrane permeability releasing cytochrome c and thereby activating caspases 9 and formation of the apoptosome. While extrinsic pathway involved, the ligand binding to death receptor triggering intracellular signals to activate caspases 8. Once executioner caspases are activated, the effector caspases come into play and form apoptotic bodies [28, 29]. Likewise, several proteins are responsible for inducing intrinsic pathway of apoptosis. The BCL-2 family and tumour suppressor proteins are of prime importance [30]. It consists of both anti-apoptotic (BCL-2 L1, BCL-XL etc.) and pro-apoptotic (BAK, BAX etc.) proteins that work in association with one another [31]. The BAK and BAX induce mitochondrial outer membrane permeabilization leading to mitochondrial dysfunction, hence marking it for apoptosis [32]. Our results also revealed the significant increase of pro-apoptotic and down regulation of anti-apoptotic genes after treated with compounds 3 (Fig. 5), suggesting the involvement of intrinsic mitochondrial pathway. The expression of p53, a tumour suppressor gene, also significantly down regulated after treatment, which gets mutated in most of the cancers and responsible for inhibiting apoptosis [33].

Angiogenesis is an important physiological process for growth and is also known to be involved in the pathogenesis of many inflammatory diseases including cancer [34]. It further strengthens and develops tumor mass by providing important nutrients and promotes migration and invasion to the secondary sites of the body [35]. Our results showed that compound 3 also inhibited the bidirectional migration to almost two folds of the NSCLC cells in a dose dependent manner by using wound healing assay. It delayed the healing process of the scratch as compared to control. It has been reported that VEGF-A (VEGF) is the key stimulator in the angiogenesis [17, 36]. VEGF binds to its receptor and triggers the intracellular signals, which lead to the initiation of a cascade of the events involved in angiogenesis. The VEGF levels increase drastically during tumour growth and contributes to enhanced stroma [37]. While COX-2, is another major enzyme responsible for converting arachidonic acid into prostaglandin H2. It is also involved in supplementing the process of angiogenesis in association with VEGF [38–40]. Thus the compounds, which can act and downregulates their expression might be an attractive target for cancer treatment [41]. Our results also showed that compound 3 significantly downregulates both the genes responsible for angiogenesis of tumour cells. They also effect the expression of matrix metalloproteinases genes (data not shown). Studies also confirmed that inhibiting COX-2 enzyme in preclinical models not only prevented angiogenesis, but also reduced the migratory and metastatic potential of tumor cells [42, 43].

## Conclusions

This current study resulted in isolation of three new sesquiterpenes namely (E)-6-(hydroxymethyl)-7-methoxy-1-(2-methylpropylidene)-1H–indene-3-carboxylic acid (1), (E)-6-acetoxy-7-methoxy-1-(2-methylpropylidene)-1H–indene-3-carboxylic acid (2) and (E)-methyl 6-acetoxy-7-methoxy-1-(2-methylpropylidene)-1H–indene-3-carboxylate (3) from ethyl acetate fraction of the aerial parts of the plant *Polygonum barbatum*. The structure elucidation and characterization of the isolated compounds 1–3 were done by using various spectroscopic techniques such as mass spectrometry, UV, IR, ^1^H NMR, ^13^C NMR and Heteronuclear multiple bond correlation spectroscopy. Among all three sesquiterpenes, compound 3 possesses the most potent anti-proliferative potential against non-small cell lung carcinoma cells (NCI-H460). It induced apoptosis and inhibited the cell migration of the cancerous cells and alters the gene expression, which is responsible for the apoptosis and angiogenesis. Thus, compound 3 can be further evaluated for its effects on the proteome by means of 2D PAGE and 2D DiGE proteomics approaches to further confirm the gene expression analysis to make it potential drug candidate.

## Methods

### General

The double focusing Varian MAT-312 spectrometer was used for EI-MS and HR-EI-MS analysis and ^1^H NMR and ^13^C NMR spectra were recorded through Bruker AMX-500 MHz Spectrometer with tetramethyl silane (TMS) as internal standard. The chemical shift values were reported as ppm and scalar coupling as Hertz (Hz). The IR spectra were recorded by using Hitachi JASCO-320-A, while Hitachi UV-3200 spectrophotometer was employed to record UV spectra. The precoated silica gel plates were used to carry out TLC and ceric sulphate in 10% H_2_SO_4_ solution was used for detection of UV active compounds. Similarly, silica gel (E. Merck, 230–400 mesh and 70–230 mesh) was used for column chromatography.

### Extraction and isolation

The whole plant of *Polygonum barbatum* (5.4 kg) was collected from Northern areas (Mansehra), Khyber Pakhtunkhwa Pakistan in October 2015. The plant was identified by Dr. Manzoor Ahmed (Taxonomist), at the Department of Botany, Government Postgraduate College, Abbottabad, Pakistan. A voucher specimen (No. 66130) has been submitted in herbarium of the same department.

The aerial part of the plant material was shade dried, ground into fine powder and extracted thrice with methanol (3 × 10 L) at room temperature and filtered. The filtrate was subjected to vacuum rotary evaporator to get crude extract (245 g). The whole extract was further partitioned into three fractions, namely *n*-hexane (85 g), ethyl acetate (48 g) and *n*-butanol (94 g).

**Fig. 8:**
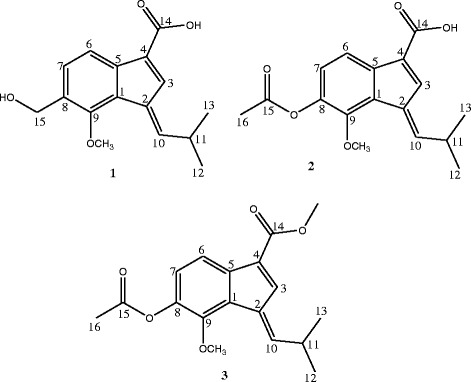
Structures of compound 1–3

The ethyl acetate fraction was chromatographed on silica gel (E. Merck, 230–400 mesh and 70–230 mesh) using the solvents with increasing polarity, *n*-hexane was used with gradient of ethyl acetate up to 100% followed by methanol, which resulted in sub-fractions depending on the polarity of compounds. Sub-fractions number 4―10 out of the total 12, were further, subjected to column chromatography to get compound 1 (8.6 mg) at EtOAc: *n*-hexane (40:60), while compound 2 (9 mg) and compound 3 (7.5 mg) were purified at EtOAc: *n*-hexane (36:64), and (27:73), respectively (Fig. 8).

### Cell culture

Cell lines (i.e., NCI-H460, MCF-7, HeLa and NIH-3 T3) were purchased from American type culture collection (ATCC, USA). Cell lines were grown and maintained using RPMI-1640 (GIBCO, Auckland, NZ) supplemented with 2 mM L­glutamine, 2 g/L D-glucose, and 1.5 g/L sodium bicarbonate, 10% heat inactivated-FBS (Hyclone, USA) and 1% antibiotic solution in a humidified (95% air: 5% CO_2_) incubator at 37 °C. The cells were regularly passaged on reaching 80% confluency using trypsin-EDTA in a t75 flask.

### MTT (3-(4,5-Dimethylthiazol-2-yl)-2,5-dipheynyltetrazolium bromide) assay

In order to evaluate the effects of the compounds on cell viability, 10,000 cells per well were seeded in a 96-well micro titer plate. After 24 h, cells were treated with various concentrations (5–250 μM) of the test compounds (1–3). After 48 h of incubation, 10 μL MTT dye (Biobasic, Canada) was added to the wells followed by 4 h of incubation. Later, the dye was removed and formazan crystals were solubilized in DMSO and absorbance was noted at 590 nm using a SpectraMax spectrophotometer (Molecular Devices, USA). The cytotoxicity at different concentrations and IC_50_ of the compounds against both cell lines were also calculated.

### Phase contrast microscopy

The effect of compound 3 on non-small cell lung carcinoma and normal fibroblast cells was evaluated under the Phase Contrast microscope (Nikon, Tokyo, Japan). Cells were grown in a 6-well plate to 60% confluency. Later, the cells were treated with compound 3 at two different concentrations (20 and 40 μM). The wells were photographed at 0, 24 and 48 h by using the microscope at 10X magnification. The images were taken immediately after the addition of compounds for 0 h as a control.

### YO-PRO-1 assay

The apoptotic potential of compound 3 was analyzed by Vybrant Apoptosis Assay Kit # 4 (Invitrogen, USA) according to manufacturer protocol. Briefly, 1 × 10^6^ NCI-H460 cells were seeded in a 6-well plate (Corning, USA). After 24 h, cells were treated with compound 3 and further incubated for 48 h in a humidified CO_2_ incubator. After incubation, the cells were washed with PBS and dissociated using trypsin-EDTA. Cell pellet was collected by centrifugation and suspended in 1 mL PBS. One microliter (1 μL) of YO-PRO-1 stock solution (Component A) and PI stock solution (Component B) were added and incubated on ice for 30 min. FACSCalibur (Becton Dickinson, USA) was used to analyze the samples whereas, CellquestPro software was used to calculate the cells in their respective phase of apoptosis. Total of 10,000 events of each sample were recorded as one reading and all experiments were performed in triplicate.

### Wound healing assay

To observe the anti-migratory effects of compound 3, cells were grown to 80% confluency in a 6-well plate (Corning, USA). A scratch was gently made using 100 μL micropipette tip. The well was thoroughly washed with 1X PBS to remove all the scratched cells and compound 3 was added. Image taken immediately was marked as 0 h reading. Scratch images were taken at 24 and 48 h by using a Phase Contrast microscope (Nikon, Tokyo, Japan). The images were processed and area of the wound was determined by using Image J software.

### RT-PCR

For the total RNA extraction, 1.5 × 10^6^ NCI-H460 cells were grown in the presence or absence of compound 3 in a 6-well plate. After 48 h of incubation, cells were washed with PBS and total RNA was extracted using TRIzol reagent (Life technologies, USA), according to the standard protocol. Extracted RNA was quantified and 1 μg RNA was used to synthesize cDNA through RevertAid First Strand cDNA Synthesis Kit (Thermo Scientific, USA), according to the user guide. This cDNA was used as template and genes related to apoptosis and angiogenesis were amplified at their respective annealing temperatures (Table 4). The final volume of PCR reaction mix was kept at 25 μL. The amplified products were resolved on 1% agarose gel. The gel was analyzed and quantified using Image J software.

**Table 4:** Primer sequences and annealing temperatures of genes used in this study

Gene	Sequence	Annealing Temperature (°C)	References
BCL-2 L1	R: ATGGTCAGTGTCTGGTCATT F: TTGTGGAACTCTATGGGAAC	57	[44]
p53	R: CTCTCGGAACATCTCGAAGCG F: GCTCTGACTGTACCACCATCC	57	[45]
BAX	R: GGCCCCAGTTGAAGTTGC F: AAGAAGCTGAGCGAGTGTC	54	[46]
BAK	R: CCTGAGAGTCCAACTGCAAA F: GGTCCTGCTCAACTCTACCC	60	[47]
VEGF	R: ACCGCCTCGGCTTGTCAC F: GTGTGCCCCTGATGCGATGCG	54	[48]
COX-2	R: CGCTCAGCCATACAGCAAATCCTT F: GTGCACTGTGTTTGGAGTGGGTTT	56	[49]
GAPDH	R: GGTCTACATGGCAACTGTGA F: ACGACCACTTTGTCAAGCTC	59	[50]

### Statistical analysis

All the results are presented as the mean ± standard deviation of triplicate experiments. Student’s t-test was used to compare the treated and control groups whereas *p* < 0.05 was reported as significant.

## Abbreviations

BCL-2: B-cell lymphoma 2
COX-2: Cyclooxygenase-2
DIGE: Difference gel electrophoresis
EDTA: Ethylenediaminetetraacetic acid
FACS: Fluorescence-activated cell sorting
FBS: Fetal bovine serum
GAPDH: Glyceraldehyde 3-phosphate dehydrogenase
HR-EI-MS: High Resolution Electroionization Mass Spectrometry
NSCLC: Non-small cell lung cancer
NSCLC: Non-small cell lung carcinoma
PAGE: Polyacrylamide gel electrophoresis
PGH2: Prostaglandin H2
PI: Propidium iodide
ROS: Reactive oxygen species
RPMI: Roswell Park Memorial Institute
RT-PCR: Reverse transcription polymerase chain reaction
TMS: Tetramethyl silane
VEGF: Vascular endothelial growth factor
